# Effect of contraction intensity on sympathetic nerve activity to active human skeletal muscle

**DOI:** 10.3389/fphys.2014.00194

**Published:** 2014-06-03

**Authors:** Daniel Boulton, Chloe E. Taylor, Vaughan G. Macefield, Simon Green

**Affiliations:** ^1^School of Science and Health, University of Western SydneySydney, NSW, Australia; ^2^School of Medicine, University of Western SydneySydney, NSW, Australia; ^3^Neuroscience Research AustraliaSydney, NSW, Australia

**Keywords:** muscle, contraction, metaboreflex, sympathetic, ischemia

## Abstract

The effect of contraction intensity on muscle sympathetic nerve activity (MSNA) to active human limbs has not been established. To address this, MSNA was recorded from the left peroneal nerve during and after dorsiflexion contractions sustained for 2 min by the left leg at ~10, 25, and 40% MVC. To explore the involvement of the muscle metaboreflex, limb ischemia was imposed midway during three additional contractions and maintained during recovery. Compared with total MSNA at rest (11.5 ± 4.1 mv^.^min^−1^), MSNA in the active leg increased significantly at the low (21.9 ± 13.6 mv^.^min^−1^), medium (30.5 ± 20.8 mv^.^min^−1^), and high (50.0 ± 24.5 mv^.^min^−1^) intensities. This intensity-dependent effect was more strongly associated with increases in MSNA burst amplitude than burst frequency. Total MSNA then returned to resting levels within the first minute of recovery. Limb ischemia had no significant influence on the intensity-dependent rise in MSNA or its decline during recovery in the active leg. These findings reveal intensity-dependent increases in total MSNA and burst amplitude to contracting human skeletal muscle that do not appear to involve the muscle metaboreflex.

## Introduction

Intermittent contraction of human skeletal muscle is accompanied by rapid vasodilation, a fall in muscle vascular resistance and a rise in muscle blood flow (Tschakovsky et al., 2004; Reeder and Green, 2012). This rapid hyperemic response is associated with an immediate fall in arterial blood pressure (Sprangers et al., 1991; Wieling et al., 1996) that is quickly damped and normalized by the baroreflex and presumed involvement of muscle sympathetic nerve activity (MSNA) (Masuki and Nose, 2003).

Theoretically, the control of the MSNA response to contracting muscle might involve central command (Matsukawa, 2012) and muscle afferent feedback (Kaufmann, 2012), as well as the baroreflex. The understanding of the control of MSNA during human exercise has been largely limited to measurements in inactive limbs (Rowell, 1993), and such measurements cannot be easily extrapolated to a contracting limb (Wallin et al., 1992). Moreover, establishing the contributions of central command and muscle afferent feedback to the control of MSNA during intermittent contractions is difficult given the profound effect of the baroreflex on MSNA (Cui et al., 2006) and the need to record sufficient bursts of MSNA for quantification within a series of short contractions.

Sustained contractions provide a slightly less complicated scenario for studying the control of MSNA during muscle contractions. This is because blood pressure does not fall at the onset of sustained contraction and, so, there is less engagement of the baroreflex, and also the more constrained movement of limbs during isometric contractions facilitates recordings to contracting muscles. To our knowledge, only two studies have recorded MSNA to active muscles during sustained contractions. Wallin et al. (1992) measured the spectral power of MSNA to dorsiflexor muscles during 3 min contractions (10–30% MVC) and reported that it decreased from resting levels and was also lower than MSNA recorded in the inactive leg at the same time. By contrast, Hansen et al. (1994) measured MSNA burst frequency to toe flexor muscles during 2 min contractions and observed no change compared with rest or MSNA in an inactive leg.

These two studies provide limited and inconsistent evidence pertaining to MSNA to a contracting muscle, perhaps partly due to differences in analytical techniques used. Other technical challenges, such as movement artifact and EMG contamination of the recorded signal, limited observations to relatively low contractile forces (5–30% MVC) and neither study explored the effect of contraction force on MSNA. Consequently, the effect of contraction force on MSNA to the active limb is not known. Therefore, the primary aim of the present study was to test the effect of contraction intensity on MSNA to active muscles. There is a perspective that chemically-mediated afferent feedback from contracting muscle, the muscle metaboreflex, is primarily responsible for the increase in MSNA during sustained and intermittent contractions (Rowell, 1993; Hansen et al., 1994). If this is true, then an ischemic stimulus added during contraction, thought to be linked to activation of the metaboreflex, should amplify the MSNA response during contraction. In addition, persistence of this ischemic stimulus after contraction should prevent the MSNA response from returning to resting levels until the stimulus is removed. Accordingly, we tested the hypothesis that the cessation of blood flow (ischemia) during and after contraction increases MSNA to contracting muscle beyond that observed during and after “control” contractions.

## Materials and methods

This study was conducted in accordance with the principles of the Declaration of Helsinki (2008) and approved by the Human Research Ethics Committee of the University of Western Sydney. All subjects provided their written, informed consent prior to participation. Eighteen subjects (12 males, 6 females; age = 18–50 years), apparently healthy and free of cardiorespiratory, metabolic or neuromuscular disease, performed a total of 22 experiments.

### Recording procedures

Participants lay semi-recumbent in a chair with their backs at 45° and their legs supported horizontally and the feet strapped to independent footplates. Spontaneous MSNA was recorded from muscle fascicles of the left common peroneal nerve supplying the ankle and toe extensor and foot everter muscles via tungsten microelectrodes (Frederick Haer and Co, Bowdoinham, ME, USA) inserted percutaneously at the level of the fibular head; a nearby subdermal electrode with a larger uninsulated tip served as the reference electrode. A suitable intrafascicular site was identified by the generation of twitches in the innervated muscle at currents <20 uA, the presence of stretch-evoked activity in muscle spindle afferents, and spontaneous bursts of cardiac-locked MSNA which increased during an inspiratory apnoea. Neural activity was amplified (gain 2 × 10^4^, bandpass 0.3–5.0 kHz) using an isolated amplifier and headstage (NeuroAmpEX, ADInstruments, Sydney, Australia) and stored on computer (10 kHz sampling) using a computer-based data acquisition and analysis system (PowerLab 16SP hardware and LabChart 7 software; ADInstruments). EMG (10 Hz–1 kHz) was recorded with Ag-AgCl surface electrodes over tibialis anterior of both legs and sampled at 2 kHz, ECG (0.3 Hz–1 kHz) was recorded via surface electrodes on the chest and sampled at 2 kHz and respiration (DC-100 Hz) was recorded using a strain-gauge transducer (Pneumotrace, UFI, Morro Bay CA, USA) wrapped around the chest and sampled at 100 Hz. Continuous non-invasive blood pressure was recorded using digital arterial plethysmography (Finometer Pro, Finapres Medical Systems, The Netherlands) sampled at 400 Hz. Dorsiflexion force was measured using two load cells (Aluminium S Type EG PT, Baulkham Hills, Australia) connected to the footplates, amplified (gain 200×, bandpass DC-10 Hz; Quad Bridge Amplifier, ADInstruments, Sydney, Australia), sampled at 100 Hz and normalized to the maximal voluntary contraction (MVC). The MVC force for each leg was taken as the highest force during 3 s maximal efforts (two per leg) performed prior to the recording of MSNA.

### Experimental protocol

**Figure 1:**
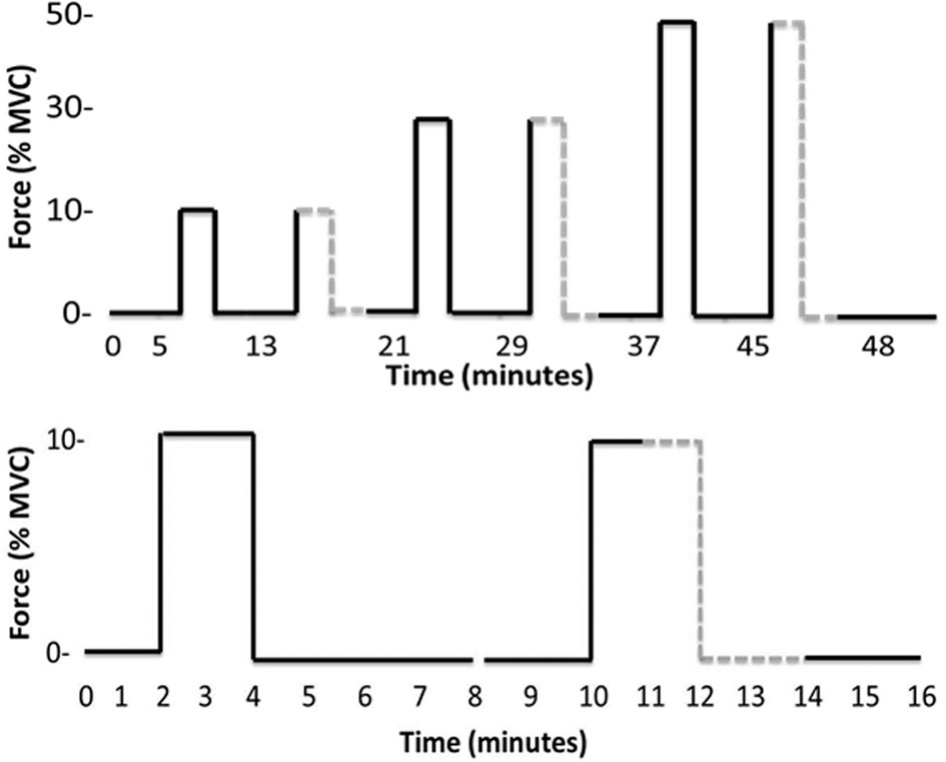
**Timelines of the entire experimental protocol (top) and the initial pair of contractions plus rest periods (bottom)**. The latter timeline more clearly identifies the onset and offset of ischemia (gray dashed line) and that each contraction belongs to an 8-min “block” beginning with a 2 min rest and ending with a 4 min rest.

Continuous recordings of MSNA were made during a 5 min baseline period followed by a 48 min protocol of isometric dorsiflexions of the ankle and intervening rest periods (Figure 1). Six contractions were made by dorsiflexors of the left leg while MSNA was recorded from this same leg. Each contraction was sustained for 2 min and preceded and followed by 2- and 4-min rest periods in an 8-min “block.” Dorsiflexions were performed at target forces of ~10, 30, and 50% MVC (Figure 1), and the actual forces are shown in Table 1. To avoid displacement of the microelectrodes, subjects were instructed to gradually increase the force of contraction to reach the target force within 10–15 s. All contractions were sustained for 2 min, at which point subjects were asked to relax quickly. To test the effect of contraction intensity on MSNA, three contractions were sustained at low, medium and high submaximal forces under *control* conditions. To explore the contribution of the muscle metaboreflex to any effect of contraction intensity on MSNA, each of these three control conditions was accompanied by identical blocks of contraction and rest periods, except *ischemia* was imposed midway during contractions and maintained for 2 min into recovery (*post-exercise ischemia*). The order of *control* and *ischemic* conditions at a given force was randomized. Limb ischemia was created by inflating a 22 cm thigh cuff to 220 mmHg within 1 s and then, 3 min later, removed with similar speed by rapidly deflating the cuff (Hokanson AG101 and E20, Bellevue, WA, USA). Ischemia was verified by absence of a pulse in the second toe of the ischemic limb, which was assessed using a piezoelectric pulse transducer attached to the toe. Since the MSNA signal deteriorated with time in some subjects, accurate MSNA data sufficient to test the experimental hypotheses were obtained in only eight subjects (6 males, 2 females).

**Table 1:** **Force, EMG, heart rate (HR), and mean arterial pressure (MAP) responses during the first and second minute of contractions at three intensities (low, medium, high) under control (C) and ischemic (I) conditions**.

		**Rest**	**Low**		**Medium**		**High**	
			**Minute 1**	**Minute 2**	**Minute 1**	**Minute 2**	**Minute 1**	**Minute 2**
Force(%Max)	C^*^^†^	0	8 ± 2	10 ± 1	24 ± 5	28 ± 3	42 ± 5	46 ± 4
	I^*^^†^	0	8 ± 2	11 ± 1	24 ± 4	29 ± 3	41 ± 4	43 ± 7
EMG (%Max)	C^*^	0	8 ± 3	9 ± 5	25 ± 14	30 ± 17	52 ± 30	58 ± 30
	I^*^	0	10 ± 6	8 ± 5	25 ± 11	24 ± 9	49 ± 20	52 ± 23
HR (bpm)	C^*^^†^[Table-fn TN3]	70 ± 10	71 ± 10	70 ± 10	73 ± 9	73 ± 10	80 ± 7	86 ± 10
	I^*^^†^[Table-fn TN3]	70 ± 11	74 ± 11	76 ± 14	74 ± 9	79 ± 10	81 ± 8	90 ± 10
MAP (mmHg)	C^*^^†^[Table-fn TN3]	95 ± 14	99 ± 11	98 ± 14	102 ± 10	102 ± 13	107 ± 10	114 ± 13
	I^*^^†^[Table-fn TN3]	94 ± 13	96 ± 10	98 ± 11	103 ± 8	108 ± 11	107 ± 9	120 ± 10

* Significant main effect of intensity (P < 0.05).

† Significant main effect of time (P < 0.05).

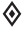
Significant intensity–time interaction (P < 0.05).

### Analysis

MSNA burst amplitudes were measured from the RMS-processed signal (200 ms moving average) using the Peak Parameters feature of LabChart 7 (ADInstruments, Sydney, Australia). In addition, spectral power of the raw MSNA signal was performed (Spectrum, LabChart 7). All statistical analyses were performed using SPSS (IBM SPSS Statistics v.21, Chicago, IL). All data were distributed normally and contrasts were made using repeated-measures ANOVA (One- and Two-Way). When significant main effects (intensity, ischemia, time) or interactions were identified, pairwise comparisons were made using a Bonferroni correction. The level of significance was set at *P* = 0.05. Results are expressed as mean ± SD (text, tables) or SE (figures).

## Results

### Resting MSNA

Rest periods preceded each of the six contractions (Figure 1) and total MSNA during the periods of rest immediately prior to each contraction ranged between 8.8 ± 5.2 and 13.3 ± 5.1 mV^.^min^−1^. There was no significant effect of time on MSNA across these rest periods, indicating that resting MSNA was stable throughout the experimental protocol.

### Effects of contraction intensity on MSNA

**Figure 2:**
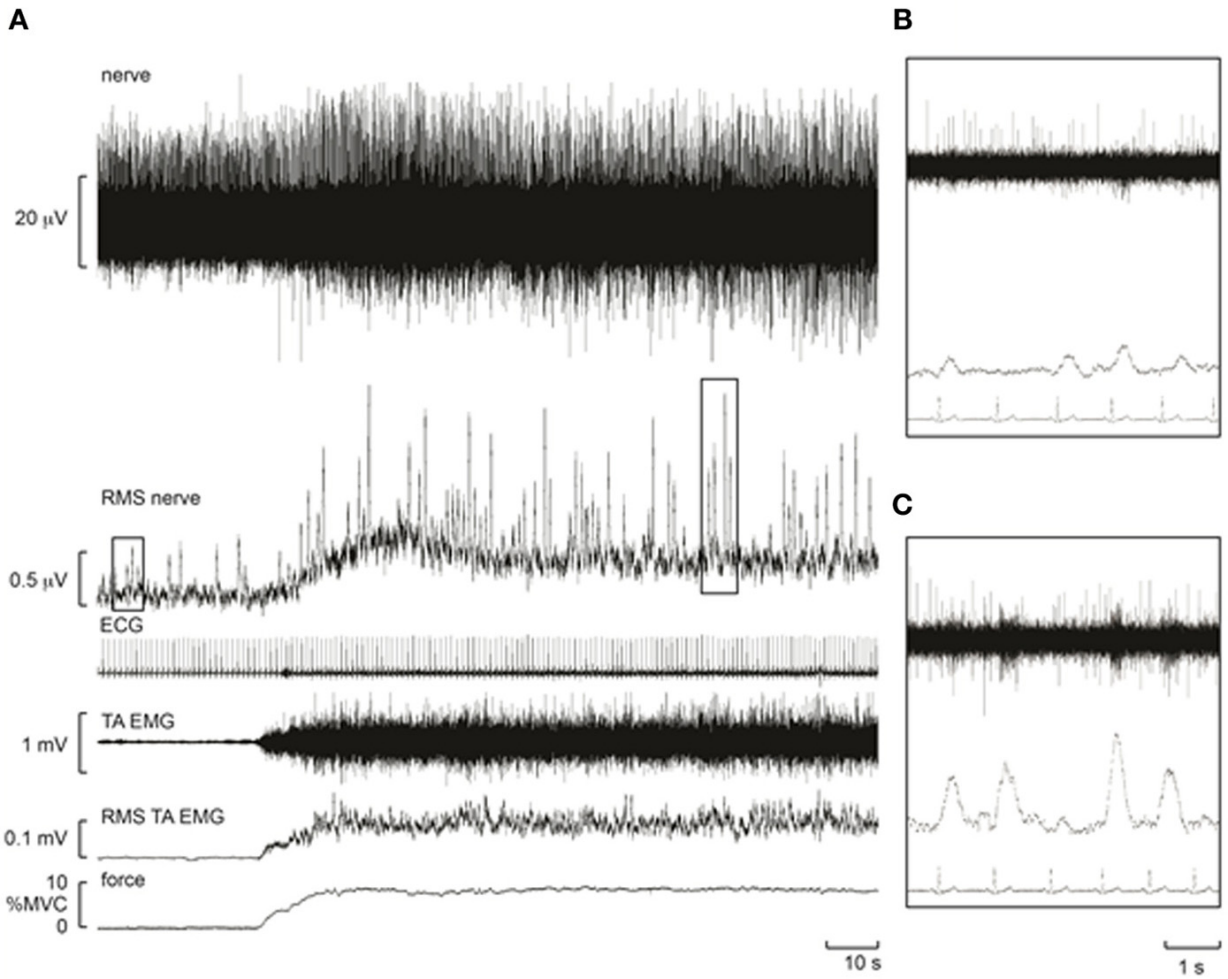
**Experimental records from one subject during a control contraction at ~10% MVC**. Note that an increase in muscle afferent activity occurs during the contraction, resulting in a baseline shift in the RMS-processed nerve signal. Despite this, bursts of MSNA remained clearly identifiable and increased in amplitude. **(A)** Raw recordings of the neurogram (top) and root mean square of this recording (“RMS Nerve”) which shows bursts of MSNA. **(B)** Zoom view of four bursts of MSNA (rms) at rest. **(C)** Zoom view of four bursts during contraction highlighting the increased amplitude of each burst compared with resting responses in **(B)**.

**Figure 3:**
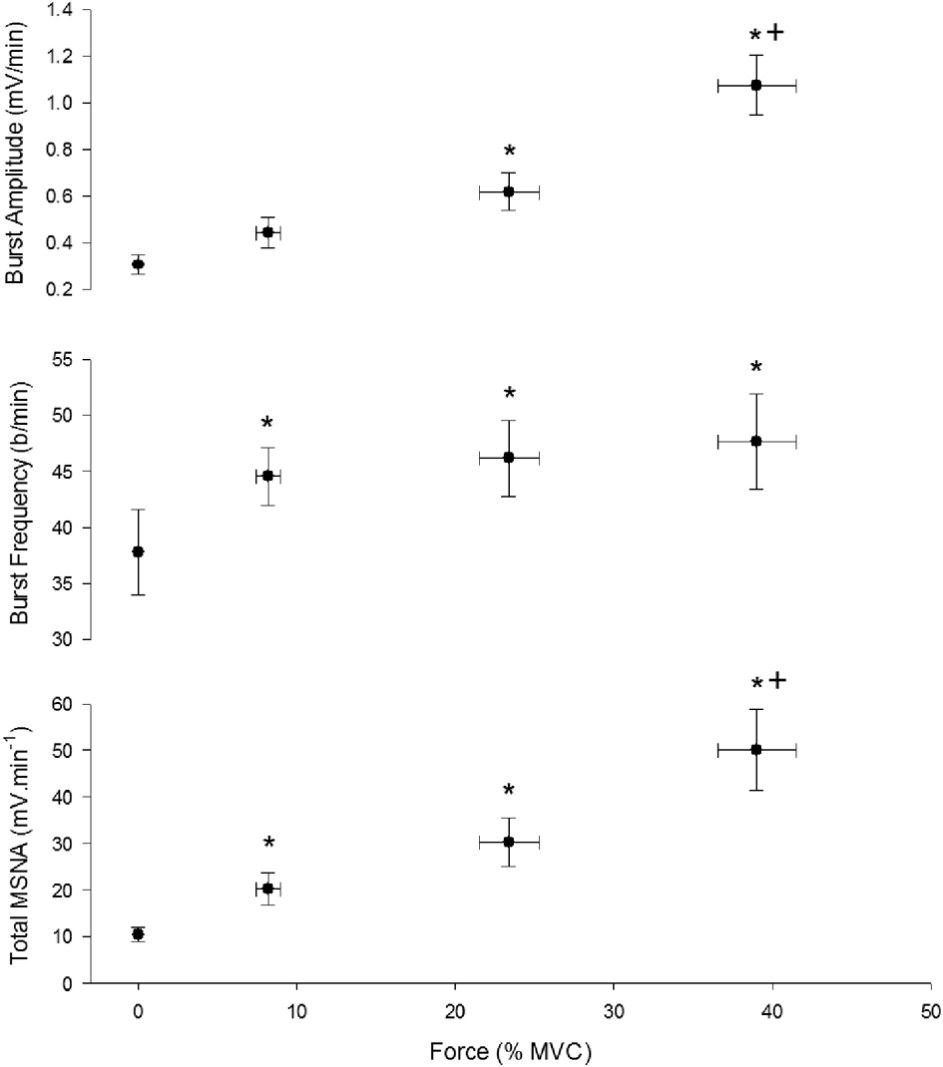
**Effect of contraction intensity on total MSNA, MSNA burst frequency, and MSNA burst amplitude during the first minute of contraction under control conditions**. There was a significant main effect of intensity on total MSNA, burst frequency and burst amplitude. Asterisk (^*^) indicates significantly different (*P* < 0.05) from the resting responses. Cross (^+^) indicates significantly different (*P* < 0.05) from 10 to 30% MVC responses at the specified time. These contrasts were based on *post-hoc*, two-tailed pairwise tests.

Three contractions were performed under control conditions and the average force, EMG, and cardiovascular responses during the first and second minute of these contractions are shown in Table 1. Experimental records from one subject performing a low-intensity contraction are shown in Figure 2. For all subjects, total MSNA during the first minute of contraction was significantly greater than at rest (*F* = 12.97, *P* < 0.001) and increased as a function of force (Figure 3). There were significant effects of intensity on burst frequency (*F* = 6.15, *P* < 0.005) and burst amplitude (*F* = 13.18, *P* < 0.001), although burst frequency failed to increase beyond the low intensity, whereas burst amplitude continued to increase in proportion to force (Figure 3). The individual recording of MSNA in Figure 2 clearly shows an increase in burst amplitude during the first minute of contraction, despite the low level of contraction. Total MSNA and burst amplitude remained elevated during the second minute of contraction and were not significantly different from the first minute, whereas there was a significant reduction in MSNA burst frequency between the first and second minute of contraction for all intensities (*F* = 8.85, *P* = 0.003).

**Figure 4:**
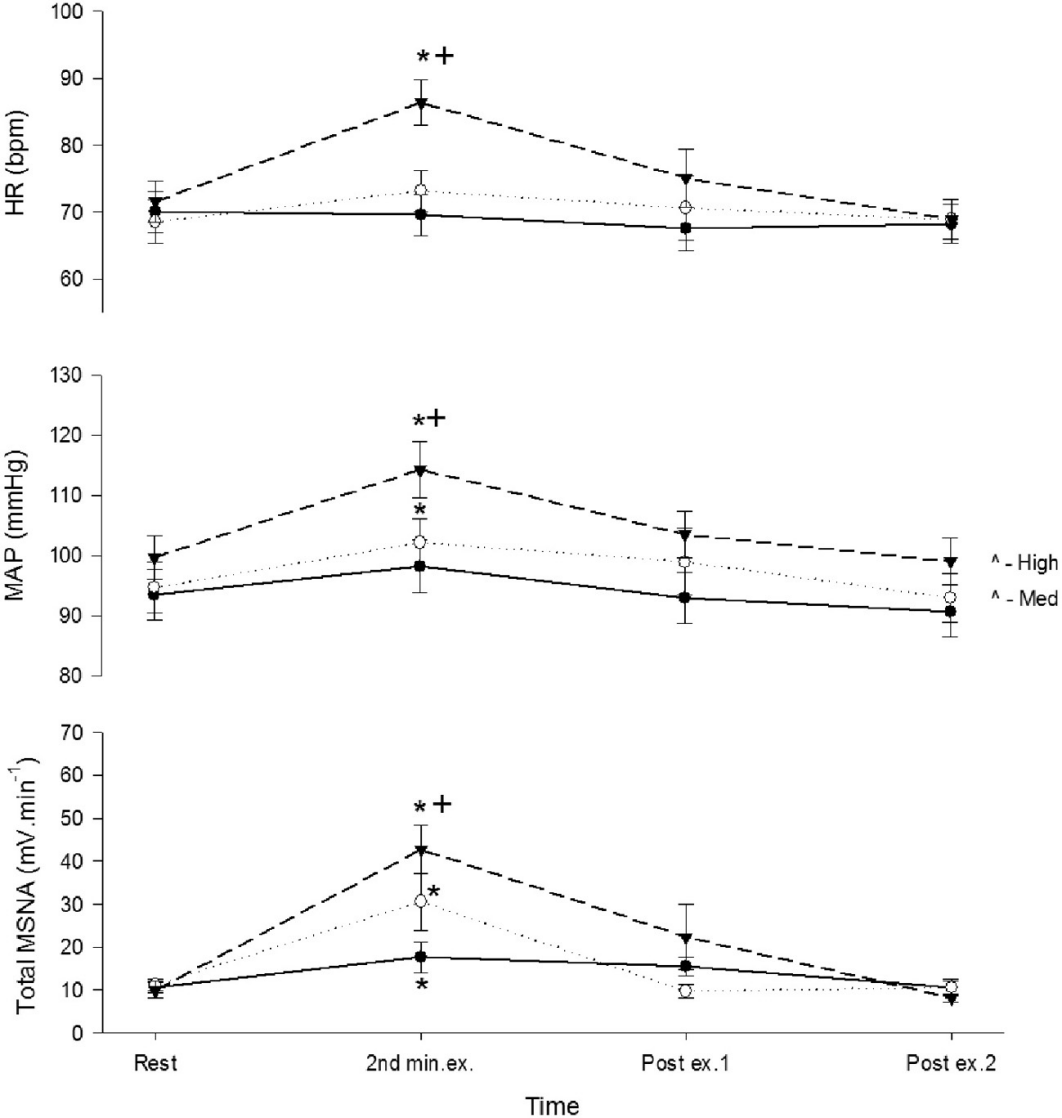
**Total MSNA, mean arterial pressure (MAP), and heart rate (HR) before, during, and after control contractions at low (closed circle), medium (open circle), and high intensities (triangle)**. Observations were made at rest, during the second minute of contraction, and during the first and second minute of recovery. Asterisk (^*^) indicates significantly different (*P* < 0.05) from the resting responses before contraction. Cross (^+^) indicates significantly different (*P* < 0.05) from the 10% MVC responses. These contrasts were based on *post-hoc*, two-tailed pairwise tests.

During recovery total MSNA, along with HR and MAP, declined rapidly during the first minute to levels not significantly different from rest immediately before contraction (Figure 4).

### Time-dependent changes in MSNA during contraction

**Figure 5:**
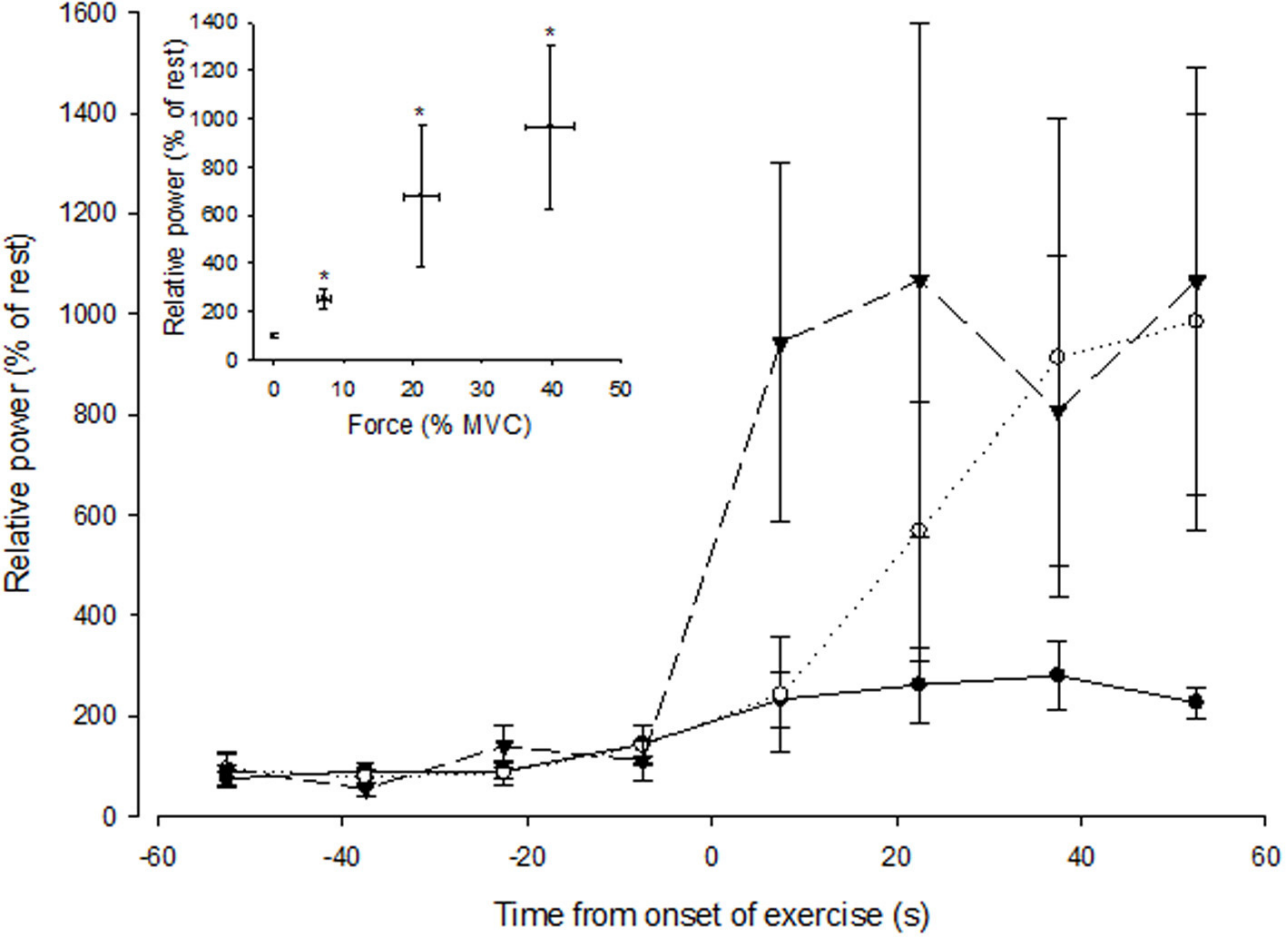
**Spectral power of MSNA (normalized to the resting value) during 15 s intervals prior to and during the first minute of contraction at low (closed circle), medium (open circle), and high (triangle) forces**. The onset of contraction is at *t* = 0 s. The inset shows spectral power of MSNA (1 min averaged values) as a function of force. The asterisk (^*^) indicates significantly different from rest at *P* < 0.05.

To explore how rapidly MSNA responded during a contraction, MSNA was analyzed during 15 s intervals prior to and during control contractions. The measurement of MSNA based on visual detection of bursts yielded more variable responses when averaged over 15 s intervals than an alternative approach based on spectral analysis of MSNA during these same periods (data not shown). Therefore, we used spectral analysis to estimate the total spectral power within the range of cardiac frequencies calculated from the ECG (R-R interval) and then normalized this estimate to the average value measured at rest during 1 min immediately prior to contraction. When averaged over 1 min, estimates of MSNA (spectral power) also showed an intensity-dependent response (Figure 5 inset; ANOVA *P* < 0.05 for main effect of intensity) and were positively correlated (*r* = 0.68, *P* = 0.07) with MSNA estimates based on the conventional approach (Figure 3). MSNA spectral power (normalized to rest) during 15 s intervals prior to and during contractions are shown in Figure 5. The effect of intensity on MSNA was evident within the first 15 s of contraction, with the normalized spectral power being significantly greater during this period than the preceding 15 s interval of rest at 50% MVC (*p* = 0.02), but not at 30% MVC (*p* = 0.06) or 10% MVC (*p* = 0.15).

### Effects of ischemia on MSNA during and after contraction

**Figure 6:**
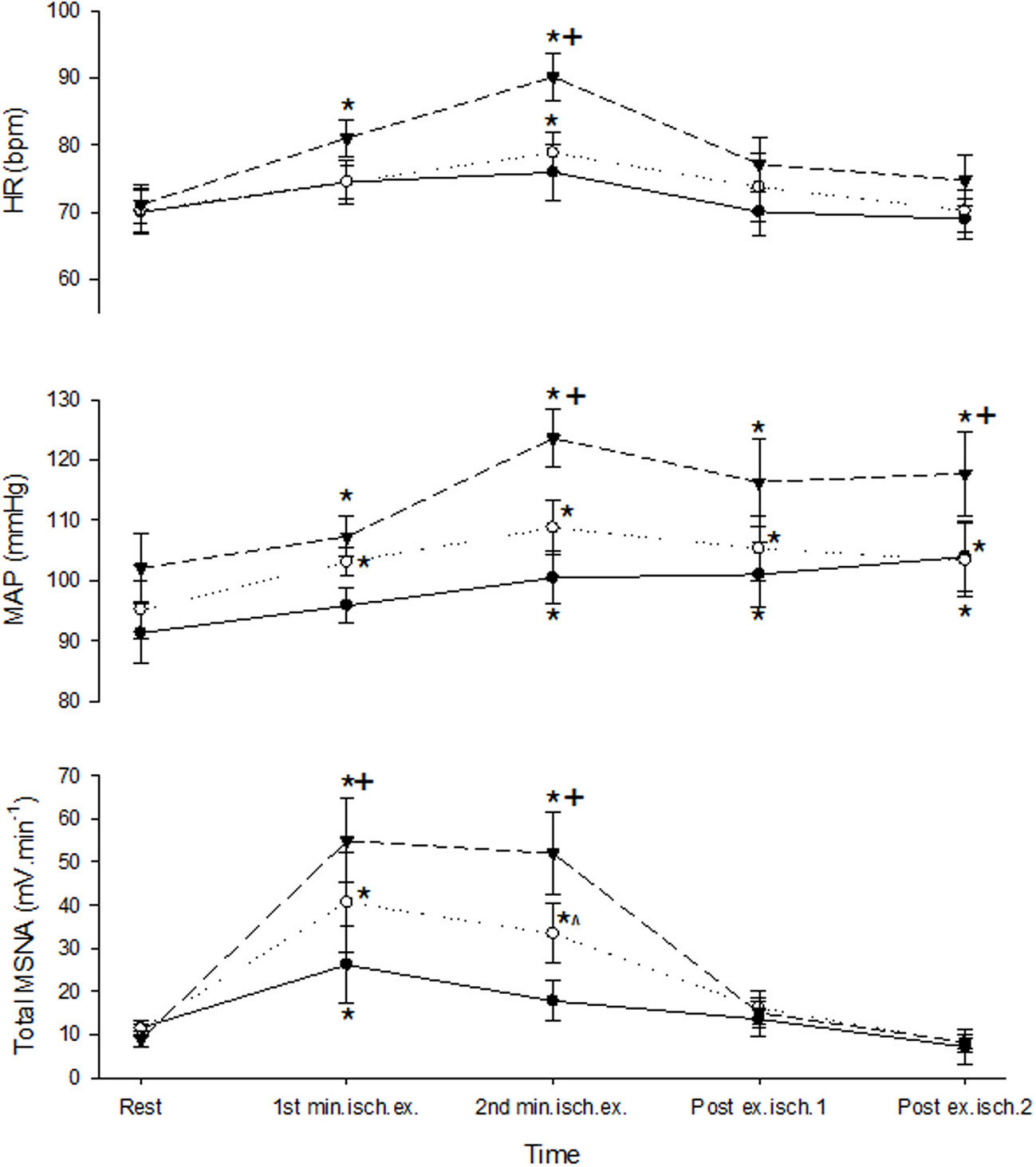
**MSNA and cardiovascular responses before, during, and after ischemic contractions at low (closed circle), medium (open circle), and high intensities (triangle)**. Ischemia was imposed at the start of the second minute of contraction and maintained during recovery. Observations were made at rest and during the first and second minutes of contraction and recovery. Asterisk (^*^) indicates significantly different (*P* < 0.05) from the resting responses before contraction. Cross (^+^) indicates significantly different (*P* < 0.05) from the 10 to 30% MVC responses at the specified time. Hat (∧) indicates significantly different from 10% MVC responses at the specified time. These contrasts were based on *post-hoc*, two-tailed pairwise tests.

A 3-min period of ischemia was imposed at the end of the first minute of contraction and persisted for 2 min beyond the end of the contraction into the recovery phase. Force, EMG and cardiovascular responses during the first and second minutes of contraction during the ischemic condition are shown in Table 1. Total MSNA responses during and after ischemic contractions and while ischemia was maintained for 2 min during recovery are shown in Figure 6. Total MSNA during the second minute of contraction (ischemia) was not different from MSNA during the first minute (non-ischemia). Total MSNA during the first and second minutes of recovery under ischemia was not significantly different from resting responses prior to each contraction (main effect of time: *F* = 2.33, *P* = 0.14, *n* = 8). MSNA burst frequency and amplitude at these same times were also not significantly different. By contrast, MAP, but not heart rate, remained significantly higher (ANOVA *P* < 0.05) during ischemic recovery (minutes 1 and 2) compared with resting, pre-contraction responses (Figure 6).

## Discussion

Understanding the neural contribution to the control of skeletal muscle vasodilation (hyperemia) and regulation of arterial blood pressure during exercise depends on knowing the effect that muscle contraction has on sympathetic (noradrenergic) nerve activity to active muscle. Prior to this study, the effect of contraction intensity on MSNA to contracting muscle was not known. For the first time, we show that total MSNA in an active limb increases in proportion to the intensity of contraction and is sustained for the duration of contraction. This intensity-dependent effect was more strongly associated with increases in MSNA burst amplitude than burst frequency. Moreover, the effect on total MSNA was rapid and observed within the initial 15 s of contraction, and was then rapidly lost when contraction ceased. These findings revealed a rapid, intensity-dependent increase in MSNA to contracting muscle, and indicated that the metaboreflex—in which MSNA remains high during a period of post-exercise ischemia—was not expressed in the active limb.

### Methodological considerations

Our observations on the effect of contraction intensity on MSNA to the active limb differ from previous findings of either no effect (Hansen et al., 1994) or a decrease in MSNA (Wallin et al., 1992). Reasons for such diverse findings are not apparent because all three studies recorded MSNA from the common peroneal nerve, subjects performed either dorsiflexion of the ankle (Wallin et al., 1992), as in the present study, or toes (Hansen et al., 1994), and the body position and intensities of contraction were very similar. Technical difficulties with performing these three studies and the frequent failure to obtain satisfactory nerve recordings means that the sample sizes are modest (*n* = 7–8) and the total number of subjects across them is small (*n* = 22). Given the very high variability of MSNA responses to a variety of stimuli (e.g., mental stress, pain) between subjects that has been reported in the literature, as well as observed in response to contraction in the present study (see SD bars in Figure 5), we cannot dismiss the possibility that the divergent findings between these three studies is linked to the sampling error that emerges from such inter-subject variability in the context of relatively few subjects. There were also differences in the way MSNA was analyzed between studies. For example, Hansen et al. (1994) reported MSNA as burst frequency, whereas the analysis in the present study based on estimates of burst frequency and amplitude suggests that burst frequency might be a less sensitive indicator of the sympathetic response to contracting muscle. Wallin et al. (1992) analyzed the spectral power of simultaneous MSNA recordings from an active and inactive leg, and the reduction in spectral power relative to that measured in the inactive leg, which was not affected by contraction, suggested that MSNA declined in the active leg. This is clearly different from the positive effect of contraction intensity on spectral power in the present study (Figure 5), and we are unable to explain this difference.

Conventional processing of the raw nerve signal enables the characteristic bursts of MSNA to be clearly identified (Figure 2), but during contractions there still remains the potential for contamination of this signal by the recruitment of motoneurons and muscle afferent activity originating in muscle spindles and Golgi tendon organs when MSNA is recorded from the active limb. Moreover, EMG from the contracting muscles can interfere with the neural signal, but this was limited by the use of high-impedance microelectrodes and locating the reference electrode close to the active microelectrode. Spectral analysis of MSNA (Wallin et al., 1992) reduces the potential contamination from EMG because the dominant frequencies of the sympathetic signal, coincident with cardiac frequencies (~1 Hz), are much lower than those of the EMG spectrum (>10 Hz). Indeed, like the time-based analysis we performed, the spectral power of the neural signal at cardiac frequencies also revealed an intensity-dependent effect of contraction on MSNA and was highly correlated with conventional measurements of total MSNA. This suggests that MSNA increases in proportion to contraction intensity and is not an artifact associated with EMG activity.

### Sympathetic outflow to contracting muscle

This is the first study to show a clear effect of contraction on MSNA to the contracting muscle that was proportional to force and with no apparent plateau. The intensity-dependent rise in MSNA in the active leg culminated in levels of MSNA at the highest intensity (~45% MVC) that were, on average, 4-fold greater than observed at rest when measured using a conventional approach, and up to 10-fold greater when using spectral analysis. The effect of intensity on MSNA was also rapid, appearing within the first 15 s of contraction, and was complemented by a rapid decline in MSNA to resting levels after contraction suggesting that the behavior of MSNA to contracting muscle is coupled closely to the onset, level and offset of contraction.

MSNA occurs in bursts synchronized to the cardiac cycle, and recent evidence suggests that the frequency and amplitude of bursts to resting muscle can be controlled independently (Steinback and Shoemaker, 2012). For the first time, the present study provides evidence of independent control of MSNA burst frequency and amplitude in a contracting human muscle. Burst frequency and amplitude increased during contraction and contributed to the increase in total MSNA in the active leg. However, the increase in burst frequency occurred only from rest to the low intensity and failed to increase beyond this level, whereas burst amplitude increased progressively and in proportion to force. This suggests that burst frequency and amplitude are differentially controlled at higher forces of contraction, and that the control of total MSNA depends predominantly on the control of burst amplitude and recruitment of additional vasoconstrictor neurons rather than by an increase in the firing probability of neurons that are already active.

### Ischemia, metaboreflex, and control of MSNA

There is a conventional view that the MSNA response during exercise is linked to activation of the muscle “chemoreflex” (metaboreflex), a view based largely on recordings of neural activity in an inactive lower limb while an upper limb is active (Rowell, 1993). Ischemia and the mismatch between metabolism and perfusion is thought to stimulate chemically-sensitive afferents in contracting muscle, activate the muscle metaboreflex, and increase MSNA to inactive regions during exercise (Mark et al., 1985; Sheriff et al., 1987; Victor and Seals, 1989; Hansen et al., 1994). This raises arterial blood pressure, and a hallmark of activation of the muscle metaboreflex is a sustained elevation in blood pressure and MSNA during post-exercise ischemia (Alam and Smirk, 1937; Mark et al., 1985; Seals and Enoka, 1989; Victor and Seals, 1989).

In the present study, ischemia (cuff inflation) was imposed midway during the 2-min contraction and continued for 2 min after contraction. At all intensities, MSNA during the second minute of contraction (ischemia) was similar to MSNA during the first minute of contraction when the thigh cuff was *not* inflated. When contraction ceased, but the cuff remained inflated and ischemia persisted, MSNA returned quickly to resting levels despite a sustained elevation in blood pressure. It is possible that this lack of effect of cuff inflation on MSNA during contraction might have been due to the relatively short period of ischemia and possible delay in activation of the muscle metaboreflex (Hansen et al., 1994), and/or an insufficient ischemic stimulus provided by cuff inflation when superimposed upon a sustained contraction which already restricts muscle blood flow (Green et al., 2011). However, the sustained elevation in blood pressure after contraction, but with the cuff still inflated, indicates that the muscle metaboreflex was activated during contraction and remained active during recovery. The fact that MSNA in active muscle declined rapidly to resting levels during this period suggests that the muscle metaboreflex was not obligatory for the contraction-induced rise in MSNA to contracting muscle. By contrast, as reported by others this reflex probably increased MSNA to *non-contracting* muscle and sustained the increase in blood pressure during the period of post-contraction ischemia (Alam and Smirk, 1937; Mark et al., 1985; Seals and Enoka, 1989; Victor and Seals, 1989).

### Control of sympathetic outflow to contracting muscle

The intensity-dependent rise in MSNA to contracting muscle could be due to increased central command, upward resetting of the baroreflex, and/or increased afferent feedback from active muscle in response to chemical and/or mechanical stimuli. Central command is of minimal importance to the MSNA response in an inactive limb (Victor et al., 1989), and it is widely thought that central command exerts its cardiovascular effects through an influence on baroreflex re-setting, vagal outflow to the heart and cardiac output (Victor et al., 1987). However, the effect of central command on MSNA to contracting muscle has not been tested, and evidence pertaining to a direct, rapid effect of central command on sympathetic outflow to the coronary vascular bed (Matsukawa, 2012) raises the possibility that central command contributes to the intensity-dependent rise in MSNA to contracting skeletal muscle. During prolonged static contractions, the baroreflex might be less engaged and contribute less to any increase in sympathetic outflow than during intermittent contractions (Rowell, 1993), but any influence of central command on MSNA to contracting muscle might also involve resetting the operating point of the baroreflex.

Afferent feedback linked to chemical and mechanical stimuli contributes to the “exercise pressor reflex.” Chemical stimuli linked to ischemia and the muscle chemoreflex, such as the increase in interstitial K^+^ (Green et al., 2000) and H^+^ concentrations (Street et al., 2001), are unlikely to contribute fundamentally to the intensity-dependent increase in MSNA to contracting muscle, but they might alter the sensitivity of afferent responses to mechanical stimuli (Kaufmann, 2012). The rapid rise and fall in MSNA at the onset and offset of contraction is consistent with a fundamental contribution by the mechanoreflex. The mechanoreflex is mediated by type III and type IV afferents (Kaufmann, 2012), and the firing frequency of these afferents varies in proportion to the contraction force and responds rapidly to contraction onset and offset (Kniffki et al., 1978; Kaufman et al., 1983; Mense and Stahnke, 1983). Activation of these afferents in human skeletal muscle by stretch or contraction also evokes a rapid, but transient, increase in MSNA in inactive muscle (Cui et al., 2006), but the effect of these manoeuvres on MSNA in contracting muscle has not been established.

### Perspective

During dynamic exercise involving many contracting muscles (e.g., walking, running, cycling), the increase in sympathetic (vasoconstrictor) outflow to several vascular beds, including *inactive* skeletal muscle, is critically important to the regulation of arterial pressure and provides a counterbalance to the vasodilation and fall in vascular resistance in contracting muscle. This sympathetic response, linked to activation of the muscle metaboreflex, does not occur at low workloads and in humans is thought to occur once the heart rate exceeds ~100 bpm (Rowell, 1993). The present findings show that sympathetic outflow to contracting muscle is increased significantly even at very low forces (<10% MVC), rises in proportion to contraction force, and does not appear to exhibit any threshold behavior. A question that arises from the present findings is why would this sympathetic outflow increase to *contracting* muscle, and in proportion to the intensity of contraction, given that such an effect opposes the need to increase muscle blood flow?

The concept of sympathetic constraint of the vasculature has been proposed in the context of *maximal* muscle blood flow and the need to prevent vasodilation from outstripping the pumping capacity of the heart during high-intensity exercise (Savard et al., 1987). Such constraint of vasodilation in contracting muscle helps regulate arterial blood pressure and serves the whole organism, similar to the effect of sympathetically-mediated vasoconstriction in some inactive regions (Rowell, 1993). The present findings, particularly the relatively large rise in MSNA at low forces, raises additional possibilities.

Despite the diversity of mechanisms involved in vascular control between organs and tissues, finely-tuned control of any vasculature in response to variation in tissue need requires both vasoconstricting and vasodilating mechanisms (Levick, 2003). No other tissue changes its metabolic need as rapidly and by as much as skeletal muscle, and its ability to vasodilate is large and rapid, particularly at the onset of low workloads (Reeder and Green, 2012). Mechanisms which help constrain this response in an intensity-dependent manner appear important. In “freely-moving” mice, failure to adequately constrain vasodilation (through sympathetically mediated vasoconstriction) at the onset of low workloads commensurate with normal foraging activity can result in an excessive fall in muscle vascular resistance and arterial blood pressure, which interrupts the continuity of such activity (Masuki and Nose, 2003). In humans, standing and being upright induces transient hypotension (Sprangers et al., 1991), and less sympathetic constraint of vasodilation in active muscles of the lower limbs could exacerbate the extent and duration of this hypotension. A rapid, intensity-dependent increase in sympathetic outflow to contracting muscle might provide an effective strategy to minimize these problems.

## Author contributions

Experiments were performed in the School of Medicine (University of Western Sydney). All authors were involved in the conception and design of the experiments, as well as the collection, analysis and interpretation of data and writing or editing of this manuscript. All authors approved the final version of the manuscript.

### Conflict of interest statement

The authors declare that the research was conducted in the absence of any commercial or financial relationships that could be construed as a potential conflict of interest.
